# Neural signals, machine learning, and the future of inner speech recognition

**DOI:** 10.3389/fnhum.2025.1637174

**Published:** 2025-07-10

**Authors:** Adiba Tabassum Chowdhury, Ahmed Hassanein, Aous N. Al Shibli, Youssuf Khanafer, Mohannad Natheef AbuHaweeleh, Shona Pedersen, Muhammad E. H. Chowdhury

**Affiliations:** ^1^Department of Electrical and Electronic Engineering, University of Dhaka, Dhaka, Bangladesh; ^2^Department of Basic Medical Science, College of Medicine, Qatar University, QU Health, Doha, Qatar; ^3^Department of Electrical Engineering, College of Engineering, Doha, Qatar

**Keywords:** inner speech recognition, inner overt speech, machine learning, deep learning, speech decoding, waves to words

## Abstract

Inner speech recognition (ISR) is an emerging field with significant potential for applications in brain-computer interfaces (BCIs) and assistive technologies. This review focuses on the critical role of machine learning (ML) in decoding inner speech, exploring how various ML techniques improve the analysis and classification of neural signals. We analyze both traditional methods such as support vector machines (SVMs) and random forests, as well as advanced deep learning approaches like convolutional neural networks (CNNs), which are particularly effective at capturing the dynamic and non-linear patterns of inner speech-related brain activity. Also, the review covers the challenges of acquiring high-quality neural signals and discusses essential preprocessing methods for enhancing signal quality. Additionally, we outline and synthesize existing approaches for improving ISR through ML, that can lead to many potential implications in several domains, including assistive communication, brain-computer interfaces, and cognitive monitoring. The limitations of current technologies were also discussed, along with insights into future advancements and potential applications of machine learning in inner speech recognition (ISR). Building on prior literature, this work synthesizes and organizes existing ISR methodologies within a structured mathematical framework, reviews cognitive models of inner speech, and presents a detailed comparative analysis of existing ML approaches, thereby offering new insights into advancing the field.

## 1 Introduction

Inner speech, also known as covert speech, is the silent, internal conversation individuals have with themselves, playing a crucial role in essential cognitive functions like planning, decision-making, and self-regulation (Alderson-Day and Fernyhough, 2015). This cognitive process involves complex neural networks that link auditory processing, motor planning, and sensory feedback, all shaped by both cognitive needs and external influences (Endicott, 2024). Mechanistic models help elucidate the neural basis of inner speech. For example, the corollary discharge model suggests that inner speech is the predicted sensory consequence of planned articulatory movements, whereas the perceptual simulation model posits that the brain reactivates stored neural patterns from past speech in regions of the brain, such as Broca’s and Wernicke’s areas (Barsalou, 2008; Scott et al., 2013; Whitford et al., 2017; Pratts et al., 2023; Gao et al., 2024). Disruptions or the absence of inner speech are observed in various disorders, including schizophrenia (Mahfoud et al., 2023), frontotemporal dementia (Vercueil, 2001), autism (Whitehouse et al., 2006; Wallace et al., 2009), aphasia (Fama and Turkeltaub, 2020), and depression (Ghamari Kivi et al., 2023). These impairments highlight the significance of accurately decoding inner speech though inner speech recognition (ISR), which could have profound implications in fields such as brain-computer interfaces (BCIs) and assistive technologies for individuals with speech impairments (Lopez-Bernal et al., 2024). Despite this transformative potential, many obstacles are associated with current technologies, including low decoding accuracy, limited generalizability across users, and insufficient real-time performance (Lopez-Bernal et al., 2024; Jahanikia et al., 2023). Thus, there is a critical need for more robust ISR systems.

Recent advances in machine learning have helped bridge the gap between cognitive foundations and practical implementations by enabling the development of systems capable of decoding inner speech from neural activity. However, effective decoding still depends on reliable signal acquisition methods. Non-invasive techniques like electroencephalography (EEG) offer high temporal resolution but suffer from low signal-to-noise ratios (Cohen, 2017), while magnetoencephalography (MEG) provides better spatial resolution but is less accessible (Baillet, 2017). Functional magnetic resonance imaging (fMRI) is mainly used for research but is limited in real-time applications due to its low temporal resolution (Logothetis, 2008; Mao, 2009). For greater precision, invasive methods like electrocorticography (ECoG) provide high-quality data, though their use is confined to extreme clinical settings (Martin et al., 2018). Preprocessing techniques, such as artifact removal, normalization, and band-pass filtering, are essential for enhancing the quality of acquired signals (Mullen et al., 2015). Once the signals are obtained, machine learning (ML) plays a pivotal role in decoding inner speech. Traditional supervised learning methods, such as support vector machines (SVMs) and random forests, have been used for feature-based classification of inner speech signals (Jahanikia et al., 2023). Recently, convolutional neural networks (CNNs) have gained prominence in inner speech recognition due to their ability to effectively extract spatial features from neural signals. CNNs excel at capturing the complex, non-linear characteristics of inner speech-related brain activity, making them highly suitable for decoding these signals with greater accuracy (Berg et al., 2021; Vorontsova et al., 2021).

Even though interest in ISR is increasing, its broader use is impeded by several fundamental scientific challenges. A significant problem is the diversity of neural representation—inner speech differs greatly among individuals regarding structure, language formulation, and timing. Such variability makes it difficult to generalize ISR models, particularly across heterogeneous user populations. Moreover, the low signal-to-noise ratio characteristic of non-invasive neural recordings such as EEG presents considerable challenges for precise decoding (Cohen, 2017; Mullen et al., 2015; Craddock et al., 2016). Neural signals associated with inner speech are frequently faint and can be easily obscured by muscle artifacts, eye blinks, and external disturbances. It is essential to surmount these challenges in order to develop robust ISR systems. ISR is particularly valuable in brain-computer interface (BCI) applications, as it provides a direct means of communication without the need for physical articulation. For people with speech impairments, like those with locked-in syndrome or anarthria, this can be life-changing: it allows for silent communication and greater self-determination (Arjestan et al., 2016; Martin et al., 2018; Lopez-Bernal et al., 2024).

In this review, we examine the current state of inner speech recognition by focusing on the various approaches used in the field across the ISR pipeline, including signal acquisition methods, preprocessing techniques, and commonly used datasets. From these existing models, we offer a comprehensive performance comparison assessing their effectiveness and practical relevance to ISR tasks. Moreover, we explain and synthesize existing machine learning approaches into a structured ISR framework aimed at clarifying the current landscape and guiding future research directions. We also discuss the limitations of current technologies and offer insights into future directions and practical applications for possible advancements.

## 2 Distinct articulatory, phonetic, and vocalic organization in inner overt speech production

In the discipline of ML, voice recognition is a broad subject that explores the subtleties of human communication, covering a range from explicit speech to the complexities of inner speech. The production of inner overt speech in terms of articulatory, phonetic, and vocalic organization is one remarkable features of this terrain as discussed below (Proix et al., 2022).

### 2.1 Articulatory variations

To make audible sounds during inner overt speech, the voice chords, tongue, and lips must move in unison. Examining the articulatory patterns involved in inner overt speech offers valuable insight into the brain mechanisms that support this process.

### 2.2 Phonetic discrepancies

The accurate articulation of phonemes, the smallest units of sound that differentiate words, characterizes the phonetic landscape of overt speech. Comprehending the phonetic characteristics of inner overt speech is critical to improving the resilience of ML models, particularly in situations where thought-based interactions or quiet communication are essential.

### 2.3 Vocalic dynamics

Pitch, intonation, and rhythm are examples of vocalic characteristics of overt speech that greatly influence how emotions and intents are expressed. The intricacies of vocalic organization in inner overt speech can be captured, offering a thorough comprehension of the complexities that ML models need to overcome to interpret the intentions encoded in speech signals.

The investigation of these unique organizational features becomes essential for the advancement of ML models in inner speech detection. Connecting the dots between spoken words’ actual physical forms and their virtual equivalents is a difficult but necessary task that could open new avenues for human-machine communication.

## 3 Related works

The investigation into inner speech has garnered scholarly attention across various disciplines, unveiling a multifaceted landscape of research endeavors aimed at comprehending the intricacies of this cognitive phenomenon. This section provides a comprehensive review of pertinent studies and scholarly contributions related to inner speech within academic discourse. This review not only serves to elucidate the diverse perspectives within the field but also lays the foundation for contextualizing the subsequent discussions on the integration of inner speech in the landscape of ML and DL for speech recognition.

Examining the literature in chronological order, (Huang et al., 2002) conducted a comparative analysis of cortical pathway activation associated with language production during both silent and overt speech. The authors suggested that the findings could have implications for aphasiology; however, they cautioned against extrapolating these findings to an aphasic population until comparable protocols are used. In a separate study, (Geva et al., 2011) executed a mixed nonrandomized control trial aimed at investigating whether individuals’ post-stroke, exhibiting impaired overt speech production, also manifest deficits in inner speech. Through the allocation of tasks, participants engaged in half using inner speech and the remaining half using overt speech, facilitating the quantification of disparities between inner and overt speech abilities. The outcomes of a Mann–Whitney test (*p* < 0.05) revealed a significant performance difference between the two groups for all three inner speech tasks, indicating that the patient group, as a whole, exhibited impairments compared to the control group.

Stark et al. (2017) conducted an experimental study involving thirty-eight individuals diagnosed with chronic aphasia (27 males, 11 females), with an average age of 64.53 ± 13.29 years and a post-stroke duration ranging from 8 to 11 months. The participants were categorized based on their speech abilities, resulting in three groups: those with relatively preserved inner and overt speech (*n* = 21), those with relatively preserved inner speech but poor overt speech (*n* = 8), and those not classified due to inadequate measurements of inner and/or overt speech (*n* = 9). The cohort, characterized by deficient overt speech, exhibited a noteworthy correlation between inner speech and both overt naming (*r* = 0.95, *p* < 0.01) and the mean length of utterances generated during a written picture description (*r* = 0.96, *p* < 0.01).

Simistira Liwicki et al. (2022) concentrated on the automated decoding of inner speech through noninvasive means, specifically EEG. The authors attained performance accuracies of 35.20 and 29.21% while classifying five vowels and six words within a publicly accessible dataset, employing the fine-tuned iSpeech-CNN architecture. Berg et al. (2021) employed a 2D Convolutional Neural Network (CNN) grounded in the EEGNet architecture. The researchers categorized EEG signals from eight subjects engaged in internal contemplation of four distinct words. The outcomes revealed an average accuracy of 29.7% for word recognition, marginally surpassing chance levels. Kiroy et al. (2022) used the Multi-layer Perceptron (MLP) neural network classification method to demonstrate accuracy in word detection within imagined speech based on brain activity patterns. The accuracy ranged from 49 to 61% for three classes and 33 to 40% for seven classes, with corresponding random recognition rates of 33.3 and 14.2%, respectively.

Nalborczyk et al. (2020) achieved a classification accuracy of 0.472 [95% CI (0.426, 0.518)] for predicting the class of nonwords during inner speech production and listening. This outcome reflects the inherent complexity and challenges in accurately classifying inner speech. In contrast, their results for overt speech production were notably higher, with a classification accuracy of 0.847 [95% CI (0.814, 0.876)]. This significant difference in accuracy between inner and overt speech accentuates the current technological limitations in decoding internal speech processes. Shepelev et al. (2021) utilized SVM and found that the average classification accuracy for the analyzed classes of speech events was relatively low, not exceeding 42.9 and 45.1%, respectively. The study also highlighted the difficulty in classifying speech intonations, with confident intonation recognized with only about 32% accuracy (±6%), and uncertain intonation detected in 48% (±5%) of cases. Neutral speech recognition was somewhat higher at 58% accuracy (±8%). These findings, while demonstrating certain limitations in current methodologies, also show the progress being made in the field. The high quality of the approaches developed by these studies suggests promising potential for future applications in BCIs, especially for those based on inner speech pattern recognition. This area of research is crucial for advancing communication technologies, particularly for individuals with speech impairments or neurological disorders. The disparity in accuracy between different types of speech and intonations also indicates the need for further research and development to enhance the effectiveness of these technologies.

The research by Arjestan et al. (2016), which probes into the development of BCIs based on decoding inner-overt speech from EEG signals, represents a significant stride in the field of speech recognition and assistive technology. This study particularly focused on developing a system that enables individuals with LIS to communicate with the external world, and to recognize overt, semi-overt, and covert speech.

The conclusion drawn from these related works emphasizes the substantial progress and diverse methodologies employed in inner speech research. Techniques like EEG, neural networks, and SVM have been instrumental in exploring the neural underpinnings and classification accuracies related to inner speech. These approaches have significantly advanced our comprehension of the complex nature of decoding inner speech patterns. Collectively, these studies lay a crucial groundwork for the field. They not only enhance our current understanding but also set the stage for future research endeavors. The insights gained from these works are pivotal in driving forward the exploration and refinement of methods in the broader scope of inner speech research. This ongoing effort is vital for the continued development of technologies that can facilitate communication for individuals with speech impairments or neurological conditions, thereby enriching their interaction with the world around them.

## 4 Data acquisition

ISR requires high-fidelity neural signal acquisition to decode the covert nature of internal speech. Selecting the appropriate signal acquisition method is crucial due to the unique trade-offs between spatial resolution, temporal resolution, invasiveness of procedure, and practicality, whereby all these factors critically influence ISR model performance.

The most widely used technique is electroencephalography (EEG) because of its excellent temporal resolution, portability, and non-invasiveness (Cohen, 2017). Particularly, these characteristics of EEG make it suitable for real-time ISR applications in both non-clinical and consumer-facing settings. Yet, along with these are disadvantages, namely low spatial resolution and high susceptibility to noise and artifacts (Hamid et al., 2021). This can reduce the precision of inner speech decoding.

As opposed to EEG, magnetoencephalography (MEG) offers better spatial localization than EEG whilst maintaining high temporal resolution (Baillet, 2017). Therefore, in the context of ISR, MEG is more effective in pinpointing the origin of brain signals related to inner speech. Despite this, many factors limit its practicality such as costliness and maintenance (Cargnelutti and Tomasino, 2023). Other factors limiting its use include the sensitivity to head movement that can distort spatial distribution, highlighting the need for stillness during recording, as well as the need for magnetically shielded environments that limit its accessibility, especially outside of research laboratory settings (Clarke et al., 2022).

Functional Magnetic Resonance Imaging (fMRI) is another neural acquisition modality that provides excellent spatial resolution (Jiang et al., 2024), whereby it is often used to map brain regions in inner speech processes. Nonetheless, fMRI is not routinely used for active ISR systems and is mainly valuable for preliminary studies and neural mapping due to the poor temporal resolution and unsuitable setup for real-time applications (Logothetis, 2008; Mao, 2009).

Electrocorticography (ECoG) is an invasive method that delivers high spatial and temporal resolution by directly placing the electrodes on the surface of the cortex (Todaro et al., 2019). Due to this direct placement, it offers the most accurate and clean signal data for ISR and has shown strong performance in speech decoding (Martin et al., 2018). However, the narrow applicability of ECoG to people in clinical settings, such as patients undergoing neurosurgery, restricts its broader use. The overview of these neural signal acquisition models used in ISR are summarized in Table 1 below.

**Table 1:** Comparative overview of neural signal acquisition methods for inner speech recognition (ISR).

Modality	Typical SNR (in dB)	Temporal resolution	Spatial resolution	Invasiveness	Practical use	ISR suitability
EEG (Berg et al., 2021; Cohen, 2017)	~0 to 5 dB (can vary widely depending on setting and task)	High	Low	Non-invasive	Portable, low cost	Real-time ISR, widely used
MEG (Baillet, 2017; Cargnelutti and Tomasino, 2023)	~3 to 10 dB	High	Medium-High	Non-invasive	Expensive, limited to labs	Useful in research
fMRI (Logothetis, 2008; Mao, 2009)	~30 to 40 dB (depends on BOLD signal quality)	Low	Very high	Non-invasive	Bulky, poor real-time performance	Brain mapping only
ECoG (Martin et al., 2018; Todaro et al., 2019)	~10 to 20 dB or higher	High	High	Invasive	Limited to mainly neurosurgical patients	High accuracy, clinical use

Based on these trade-offs, EEG remains the most commonly used practical modality for ISR, whereas other methods like MEG, fMRI, and ECoG are often limited to specialized research or clinical contexts. Thus, this makes the effectiveness of ML and DL models in ISR highly dependent on meticulous data acquisition procedures. Given the inherently covert and internal nature of inner speech, distinct challenges arise, necessitating methodological precision in acquiring pertinent data for both model training and evaluation.

The primary limitations evident in extant research pertain not only to reproducibility and the accessibility of data and code, but also to the consistency and quality of neural data acquisition across diverse populations. In a seminal study, (Stark et al., 2017) undertook an experiment involving thirty-eight individuals afflicted with chronic aphasia. Rigorous demographic profiling, encompassing gender distribution, average age (64.53 ± 13.29 years), and time elapsed since stroke (8–11 months), was meticulously executed. This demographic information assumes critical significance in gauging the generalizability of ISR models across heterogeneous populations. Acknowledging the nuanced spectrum of inner and overt speech capabilities, participants were systematically categorized into distinct cohorts. By de-alienating individuals exhibiting relatively preserved inner and overt speech, those manifesting relatively preserved inner speech with concurrent poor overt speech, and a subgroup eluding classification due to inadequate measurements of inner and/or overt speech. Such meticulous stratification facilitates model tailoring to specific subpopulations, recognizing the intricacies of inner speech attributes.

Diverse screening techniques and preprocessing procedures have been employed on the data. Preprocessing is paramount for ISR as it plays a critical role in ensuring the fidelity of neural signals (Mullen et al., 2015). As previously mentioned, EEG signals have high susceptibility to various sources of noise, and so if these interferences are not addressed, these artifacts can obscure the subtle neural signatures of inner speech and lead to a higher frequency of false negatives or positives (Ingolfsson et al., 2022). The choice of specific preprocessing techniques in the reviewed literature is mainly attributable to the nature of EEG data along with the challenges of inner speech. For instance, (Nieto et al., 2022) implemented a data filtering process within the frequency range of 0.5 to 100 Hz, where this was done to isolate frequencies relevant to cortical activity while excluding irrelevant low-frequency drift and high-frequency noise. Along with this, a 50 Hz notch filter was used to eliminate powerline interference. Additionally, an Independent Component Analysis was utilized to identify and eliminate noise-afflicted components, predominantly those contaminated with ocular and muscular artifacts. EEG, electrooculography (EOG), and electromyography (EMG) data were obtained through a BioSemi ActiveTwo high-resolution biopotential measuring system. With regards to the EEG signals, they were recorder using 128 scalp electrodes and 8 external sensors for eye and muscle activity, with high resolution and a sampling rate of 1,024 Hz. On the other hand, in the investigation conducted by (Kiroy et al., 2022), continuous EEG recordings were obtained monopolarly from 14 channels (f3, f4, f7, f8, Fp1, Fp2, c3, c4, t3, t4, t5, t6, p3, p4), following the international 10×20 system arrangement. The recordings were facilitated using the “ENCEPHALAN 131” amplifier, manufactured by “MEDICOM-MTD” in Taganrog, Russia. In brief, careful electrode placement is imperative for minimizing EOG contamination and improving spatial localization.

In order to improve signal quality for inner speech recognition (ISR), contemporary EEG (electroencephalography) preprocessing combines deep learning and adaptive learning techniques.

### 4.1 Adaptive filtering

Nonstationary noise, such eye blinks and muscular movement, is adaptively suppressed using methods like Least Mean Squares (LMS) and Recursive Least Squares (RLS). A new hybrid technique that included adaptive filtering with ICA greatly enhanced artifact removal while maintaining the integrity of the cognitive signal (Kher and Gandhi, 2016).

### 4.2 Wavelet + ICA

Wavelet denoising and Independent Component Analysis (ICA) work together to preserve neuronal characteristics while reducing transient and structural artifacts (Veeramalla et al., 2025).

### 4.3 Adversarial denoising (GAN/WGAN-GP)

In artifact-heavy EEG recordings, Generative Adversarial Networks (GANs) and Wasserstein GAN with Gradient Penalty (WGAN-GP) have demonstrated up to 14.5 dB gains in signal-to-noise ratio (SNR), surpassing conventional denoising (Tibermacine et al., 2025).

### 4.4 pix2pix autoencoder GAN

This design efficiently eliminates EMG noise and produces a high-fidelity reconstruction of a clean EEG (Wang et al., 2024).

More reliable and real-time inner speech decoding is made possible by these preprocessing techniques, which range from deep adversarial frameworks to adaptive filters. This is essential for real-world brain-computer interface (BCI) applications.

Five right-handed male subjects aged 25–31 participated in the study Arjestan et al. (2016) and EEG signals were recorded using a SAM25FO system with 21 active Ag–AgCl electrodes. A head-cap was used to position the 21 EEG electrodes on the scalp according to the international 10–20 system. The ground electrode was placed at Fpz, and the right mastoid was used as a reference. Fp1 and Fp2 electrodes were not used due to high EOG noise, whereby this choice underscores the importance of removing high-noise channels to preserve signal integrity. On the other hand, by the experimental protocol of Lee et al. (2021a), EEG signals were recorded in response to speech stimuli and resting periods, with a total of 300 trials for each condition.

The acquisition of inner speech data mandates the application of sophisticated measurement techniques. It requires exhaustive assessments, including an exploration of the interplay between inner speech and overt naming, as well as an analysis of the mean length of utterances during a written picture description. These refined measurements contribute not only to a nuanced comprehension of inner speech dynamics but also furnish indispensable data for the training of ML algorithms. In Martin et al. (2018), subdural electrode grids implanted during the surgical procedures recorded ECoG signals. These grids were made of platinum-iridium and spaced 0.6 to 1 cm apart. Thorough statistical scrutiny assumes paramount importance in deriving meaningful insights from the acquired data. Some authors have employed correlation coefficients to elucidate significant relationships, providing insights into the strength and directionality of associations between inner speech and overt naming, as well as mean length of utterance. Stringent significance thresholds (*p* < 0.01) were established to fortify the robustness of the findings (Martin et al., 2018).

One of the main concerns in inner speech research is when incomplete data prevents some participants from being properly classified. It is imperative to transparently acknowledge and address this limitation, underscoring the imperative for future investigations to adopt comprehensive data acquisition strategies. The fastidious acquisition of inner speech data necessitates discerning participant recruitment, meticulous stratification protocols, sophisticated measurement methodologies, and rigorous statistical analyses. These considerations collectively underpin the construction of precise and dependable ML and DL models for ISR.

## 5 Datasets used

The investigation into ISR within the context of ML and DL methodologies necessitates a rigorous examination of the datasets employed. The selection and characterization of datasets play a pivotal role in shaping the robustness and generalizability of models developed for this intricate cognitive process. In this section, we provide a comprehensive overview of the datasets utilized in the reviewed literature, highlighting key considerations such as screening methodologies, preprocessing techniques, and the overall data landscape. This exploration aims to elucidate the foundations upon which subsequent analyses and model development have been built, offering insights into the challenges and opportunities inherent in leveraging diverse datasets for advancing our understanding of inner speech within the computational paradigm.

EEG stands as a widely adopted modality for analyzing inner speech and open access EEG datasets are frequently utilized in studies. The dataset compiled by (Pressel Coretto et al., 2017) comprised information provided by fifteen young adults who volunteered to take part in the study. EEG signals were systematically recorded under two distinct conditions: during instances of inner speech and pronounced speech. These specific conditions were chosen strategically to facilitate subsequent investigations aimed at discerning EEG patterns distinguishing overt from covert speech. Each participant conducted 50 trials, consisting of repetitions distributed across various blocks. Among these, 40 trials corresponded to the imagined speech mode, while the remaining 10 were representative of the pronounced speech modality. The dataset by Nieto et al. (2022) comprises information from ten participants obtained under the specified paradigm, alongside two related paradigms, utilizing an acquisition system equipped with 136 channels. Jones and Voets (2021) employed an unusually extensive dataset of 7 T functional magnetic resonance imaging (fMRI) to train a deep neural network (DNN). This dataset was acquired as a single healthy volunteer engaged in multiple hours of covert reading and repetition tasks. Shepelev et al. (2021) conducted a series of psychophysical experiments to establish a voice database. The experimental cohort comprised 12 healthy female second-year bachelor students, with a mean age of 19.6 ± 0.8 years.

The dataset used in Arjestan et al. (2016) comprises EEG signals recorded from five male subjects, focusing on imagined speech tasks. Three protocols were employed: overt speech without vibration of the vocal cords, semi-overt speech (vocal track forming without pronouncing), and covert (silent) speech. The dataset includes three syllables (/kaː/, /fiː/, and /suː/), six vowels (/æ/, /e/, /au/, /aː/, /iː/, and /uː/), and resting states in Persian. Nine subjects, including three males with an average age of 25.00 ± 2.96, participated in the study by Lee et al. (2021a) and the dataset used in the study (Martin et al., 2018) involved ECoG recordings obtained from seven patients undergoing neurosurgical procedures for epilepsy, all of whom provided informed consent.

In light of the studies discussed above, we have delineated the specific datasets employed in the respective studies, shedding light on the meticulous processes involved in data acquisition. The utilization of comprehensive datasets, whether through extensive fMRI recordings, EEG channels, or psychophysical experiments, reflects the dedication to robust empirical foundations within the examined research endeavors. These datasets, meticulously curated and selected, serve as the cornerstone for subsequent analyses and model training in the pursuit of advancing our understanding of various aspects related to speech, cognition, and neural processes. As we move forward in this review, the diversity and depth of the datasets underscore the significance of methodological choices and contribute to the richness of insights derived from the collective body of research in the field of speech recognition and cognitive sciences.

## 6 Organizing existing frameworks in ISR

In the continuous evolution of speech recognition technologies, the integration of DL and ML frameworks stands as a pivotal frontier, particularly within the intricate domain of ISR. This section delves into the cutting-edge methodologies proposed by researchers to harness the power of neural networks and ML algorithms for deciphering the complexities of inner speech. From novel model architectures to refined training strategies, this exploration serves as a glimpse into the forefront of research endeavors that strive to bridge the gap between the intricacies of human cognition and the capabilities of artificial intelligence (AI) in the realm of speech recognition.

### 6.1 Mathematical formulations

The application of DL and ML frameworks in ISR draws upon well-established mathematical models capable of discerning and interpreting the intricate patterns inherent in inner speech data. This section elucidates the mathematical foundations that underpin commonly used frameworks, providing insight into the methodologies employed to bridge the gap between raw data and meaningful insights.

**Figure 1:**
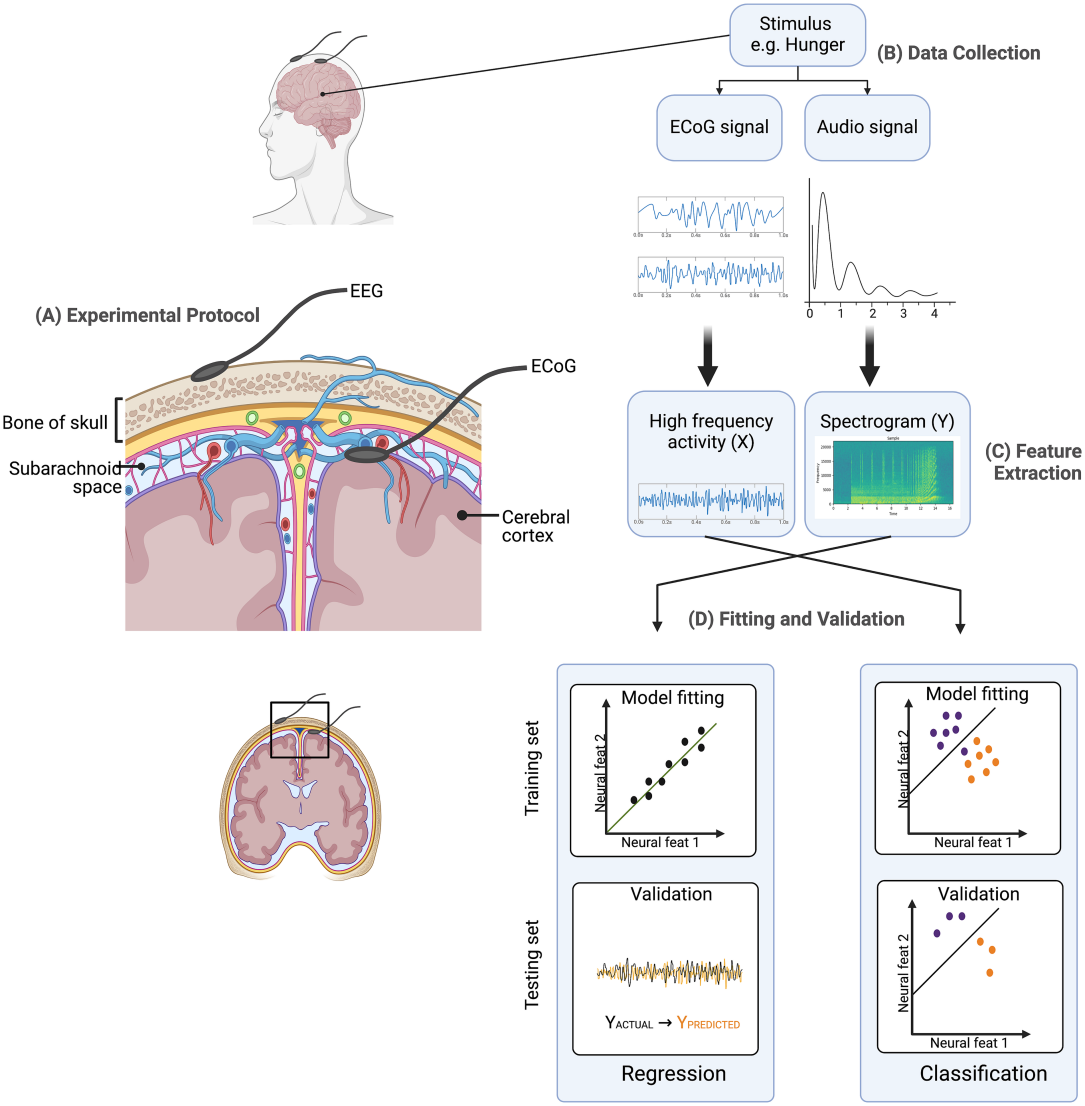
Predictive model overview. **(A)** Experimental Protocol: Electroencephalography (EEG) electrodes are placed on the scalp, while electrocorticography (ECoG) electrodes are placed on the cortical surface beneath the skull to capture neural signals. **(B)** Data Collection: Neural signals (ECoG) and corresponding audio signals are recorded in response to stimuli (e.g., hunger). **(C)** Feature Extraction: Neural data are processed to extract high-frequency activity (X), while audio data are converted into spectrograms (Y). **(D)** Fitting and Validation: Extracted features are used to train and validate models using both regression and classification frameworks. Training and testing sets are utilized to assess model performance.

A frequently employed modeling methodology involves adopting a regression framework to establish a connection between brain activity and a stimulus or mental state representation. Specifically, the stimulus features at a particular time are conceptualized as a weighted sum of neural activity, expressed as follows:


Y(t)=∑w(p).X(t,p)

where Y (t) is the stimulus feature at time t, X(t, p) is the neural activity at time t and feature p, w(p) is the weight for a given feature p (Martin et al., 2018). Another prevalent decoding model is classification, where neural activity is categorized as pertaining to a discrete event type from a finite set of choices. Both modeling approaches, regression, and classification, can employ a spectrum of ML algorithms. These algorithms encompass elementary regression techniques to more intricate non-linear methods, including hidden Markov models, support-vector algorithms, and neural networks etc. as shown in Figure 1.

Decoding models enable researchers to leverage brain activity for inferring the stimuli and/or experimental properties most likely present at each moment in time. The decoder serves as a proof of concept: when presented with a novel pattern of unlabeled brain activity (i.e, brain activity without its corresponding stimulus properties), it has the potential to reconstruct the most probable stimulus value that elicited the observed brain activity (Pasley et al., 2012).

feature $(t)=\sum_{j}^{N_{\text{lags}}}\;\sum_{i}^{N_{\text{channels}}}\;{\text{activity}}_{i}{\left(t+j\right)}^{*}$ weight ${\;}_{i,j}+\text{error}(t).$

In vector notation, this is represented as follows:


s=Xw+∈

In vector notation, the expression is characterized by vectors, encompassing stimulus feature values observed chronologically, and the matrix X representing channel activity. Each row of X corresponds to a specific time point, and each column denotes a neural feature, with separate columns accounting for time lags. The vector w comprises model weights, with each weight corresponding to a neural feature*time lag combination. Additionally, ǫ signifies a vector representing random noise at each timepoint, commonly assumed to follow a Gaussian distribution.

In conclusion, this section consolidates existing mathematical frameworks used in ISR studies, offering insight into current modeling approaches and identifying areas for optimization. The constant pursuit of more efficient and accurate mathematical frameworks remains paramount, propelling the evolution of ISR technologies and contributing to the broader intersection of cognitive science and AI.

### 6.2 Architectural frameworks in ML and DL for ISR

Analyzing model structures is essential for enhancing ISR using ML and DL techniques. This section navigates through a spectrum of architectural frameworks, drawn from existing ISR literature, and highlights how these models distill the intricate patterns inherent in inner speech data. Rooted in a foundation of computational elegance and cognitive insights, the discussion unfolds around innovative structures, model complexities, and their theoretical underpinnings. The overarching aim is to discern the nuances and overall potential offered by various architectural paradigms in the realm of ISR. As we delve into this academic discourse, the emphasis is on providing a comprehensive survey and critical analysis of architectural choices, contributing to the scholarly dialogue surrounding the effective fusion of ML and DL techniques for decoding the complexities of inner speech. The various ML and DL models used for detecting inner speech in reviewed papers are discussed in Table 2 below.

**Table 2:** Proposed ML and DL models for inner speech recognition.

Article	Model	Approach	Sample size	Class set size	Public availability
Simistira Liwicki et al. (2022)	q (CNN)	Models are able to analyze the EEG data and identify patterns and features related to inner speech. The authors highlight the subject-dependent and subject-independent approaches in using these models for inner speech decoding.	15	11 (5 vowels: /a/, /e/, /i/, /o/, /u/; 6 words: arriba/up, abajo/down, derecha/right, izquierda/left, adelante/forward, atrás/backwards)	Yes (available at: https://github.com/LTU-Machine-Learning/Rethinking-Methods-Inner-Speech)
	Gated recurrent unit (GRU)				
	Long short-term memory networks (LSTM)				
Nieto et al. (2022)	Extreme learning machines (ELM)	The training procedure of an Extreme Learning Machine (ELM) involves two distinct steps. Initially, the input weight matrix (W) and the bias weight vector (b) are randomly initialized as independent realizations, typically drawn from a uniform distribution. Subsequently, the second step entails determining the suitable output weights (beta) using the Moore-Penrose generalized inverse (Haltmeier et al., 2024).	10	4 words (arriba, abajo, derecha, izquierda, i.e., “up,” “down,” “right,” “left”)	Yes (available at: https://doi.org/10.18112/openneuro.ds003626.v2.1.0)
Berg et al. (2021)	EEGNet	EEGnet represents a compact convolutional neural network specifically crafted for diverse EEG-related classification endeavors. It demonstrates an aptitude for capturing prevalent temporal and spatial EEG features through the application of its convolutional filters.	8	4 words (up, down, left, right)	Yes (available at: https://doi.org/10.18112/openneuro.ds003626.v2.1.0)
Jones and Voets (2021)	Deep neural network (DNN)	Network weights underwent optimization through maximum likelihood estimation, employing stochastic gradient descent with Nesterov momentum. The objective function utilized a cross-entropy loss. Each layer’s weights were initialized from a Xavier uniform distribution. Input features were standardized using the mean and standard deviation derived from the training data.	1	9 target syllables (/ga/, /gi/, /gu/, /ma/, /mi/, /mu/, /sa/, /si/, /su/)	Not available
	Support vector machine (SVM)	The control analysis was trained on 50 self-generated data points using leave-one-out cross-validation, resulting in 49 training examples for each train-test split. Notably, it outperformed DNNs in test accuracy with limited inner speech data.			
Kiroy et al. (2022)	Support vector machine (SVM)	Achieved a notable level of identification and discrimination of the resting state. This success was particularly evident in models utilizing non-linear kernels, such as the sigmoid and radial basis function (RBF).	10	6 words (up, down, right, left, forward, backward)	Not available
	Multi layer perceptrons (MLP)				
Geraci et al. (2021)	Bayesian multilevel linear model (BMLM).	Models were fitted using the BRMS package with weakly informative priors. Two Markov Chain Monte Carlo (MCMC) runs were executed for each model to approximate the posterior distribution, consisting of 5,000 iterations each with a warm-up phase of 2,000 iterations.	Not applicable	Not applicable	Not applicable
Shepelev et al. (2021)	Neural network	Two computational approaches were tested for recognizing implicit speech intonations (C/U/N): generalized and personalized. In the generalized approach, classifier parameters were determined during the training session, and the model was subsequently tested with the validation sample of the second group’s speech recordings. In the personalized approach, optimal parameter values were calculated individually for each participant.	12	3 classes: Confident (C), Uncertain (U), Neutral (N)	Not available
Stephan et al. (2020)	A general linear model (GLM)	Utilizing a canonical Hemodynamic Response Function (HRF) yielded Beta-values for each condition (inner/overt), each channel, and each hemoglobin (oxy, deoxy). These values were then utilized for subsequent statistical analyses. The fNIRS data were ultimately averaged across participants.	46	2 (Inner speech, overt speech)	Not available

The exploration of proposed ML and DL models within this section underscores the versatile and innovative approaches employed in decoding inner speech. From neural network architectures to advanced training strategies, the methodologies discussed demonstrate a concerted effort to enhance the accuracy and efficiency of ISR systems. The amalgamation of computational techniques with cognitive insights not only reflects the interdisciplinary nature of this field but also underscores the potential for transformative advancements. As we move forward, these proposed models serve as a foundation for continued exploration and refinement, offering valuable contributions to the evolving landscape of ISR through ML and DL methodologies.

Interpretability is still a major concern, especially for clinical applications like speech brain-computer interfaces (BCIs), even though Table 2 compares traditional machine learning (ML) models (e.g., Support Vector Machines, Extreme Learning Machines) and deep learning (DL) architectures (e.g., CNN, EEGNet, LSTM) in the context of inner speech recognition. For BCI users who depend on these systems for mobility or communication, the “black-box” nature of DL models may make it difficult to understand the reasoning behind some classifications, which could result in misclassification concerns (Gandin et al., 2021).

In order to display feature importance and obtain understanding into decision limits, recent explainable AI (XAI) projects have created frameworks like SHAP (SHapley Additive exPlanations) and LIME (Local Interpretable Model-Agnostic Explanations). Although it was not included in their study, Simistira Liwicki et al. (2022) point out that saliency maps could be added to CNNs to improve interpretability. In order to guarantee openness and user confidence, future research should concentrate on integrating XAI approaches into BCI pipelines.

## 7 Synthesis of methodological approaches in ISR

Within the growing intersection of ML and DL, this section synthesizes existing methodologies applied in ISR research tailored for the nuanced investigation of inner speech. The discourse unfolds across a spectrum of proposed approaches, each strategically employing ML and DL techniques to discern and interpret the intricacies inherent in ISR. This scholarly endeavor focuses on reviewing representative strategies, model architectures, and training paradigms, collectively contributing to the ongoing advancement of sophisticated systems designed to navigate the complex fabric of inner speech. Grounded in both neuroscientific insights and computational progress, the methodologies presented herein represent a concerted scholarly effort aimed at bridging the cognition and AI realms, offering valuable insights into the transformative potential of ML and DL in unraveling the complexities of inner speech.

**Figure 2:**
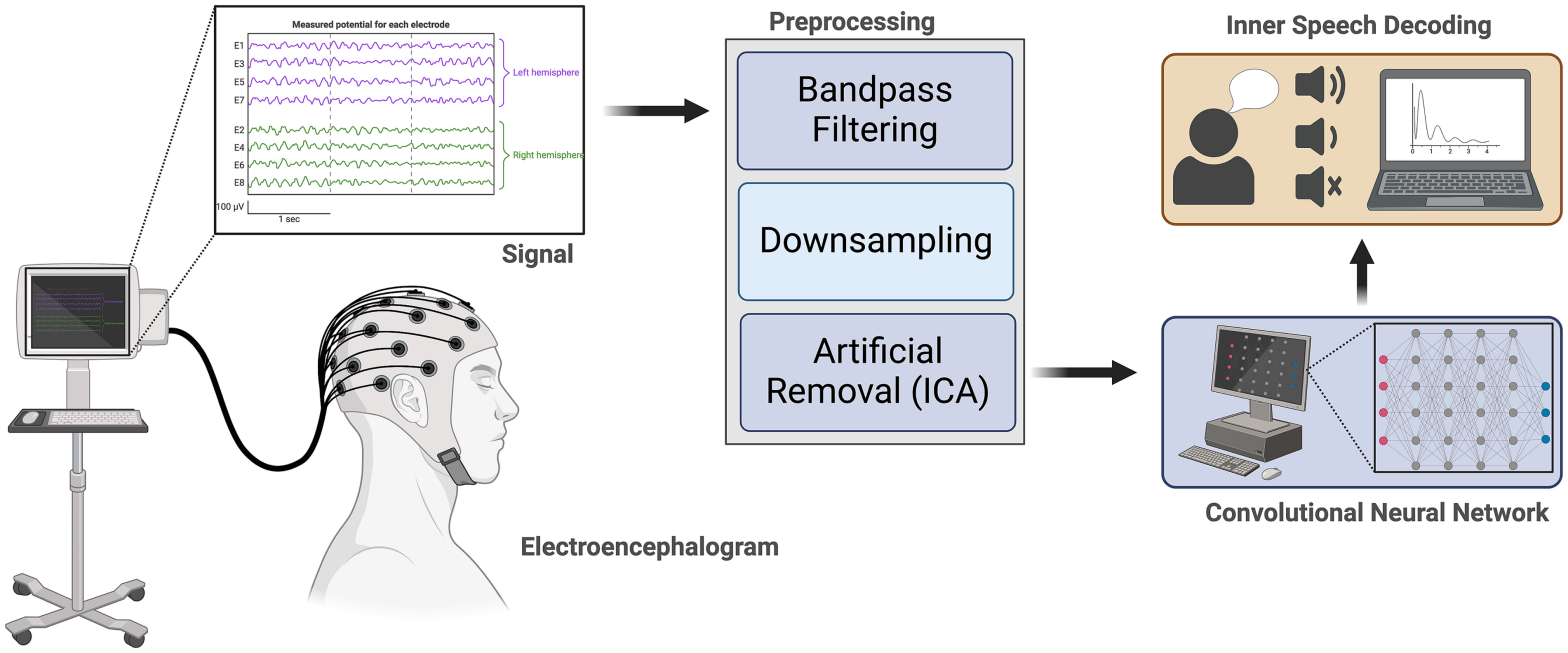
Representation of Inner Speech Recognition Methodology. *Signal acquisition:* Electroencephalography (EEG) signals are recorded from scalp electrodes, with representative traces shown for electrodes over the left and right hemispheres. *Preprocessing:* Acquired EEG signals undergo bandpass filtering, down sampling, and artifact removal using independent component analysis (ICA) to enhance signal quality. *Feature extraction and decoding:* Preprocessed signals are input to a convolutional neural network (CNN) for feature extraction and classification. *Inner speech decoding:* The CNN output is used to decode inner speech, distinguishing between different speech-related mental states and visualizing results on a computer interface.

Simistira Liwicki et al. (2022) addressed the challenge of detecting five vowels and six words using a publicly available EEG dataset. Figure 2 illustrates the workflow of the proposed approach, wherein distinct networks are trained for vowels and words, guided by the architecture depicted in the same figure. The proposed network draws inspiration from the work of Cooney et al. (2020).

Feature extraction and classification represent the two primary facets of the methodologies discussed in the context of ISR. Feature extraction is pivotal as it involves the identification and extraction of pertinent information or patterns from the raw data, thereby capturing the intrinsic characteristics of inner speech signals. This step is critical for transforming complex input data into a more manageable and informative representation. On the other hand, classification is the subsequent process, wherein the extracted features are utilized to categorize or label the inner speech data into predefined classes, such as specific speech sounds or spoken words (Geva et al., 2011). The effectiveness of the overall methodology hinges on the synergy between these two components, where robust feature extraction lays the foundation for accurate and discriminative classification, collectively contributing to the advancement of ISR systems.

### 7.1 Feature extraction

The extraction of discriminative features constitutes a pivotal stage in the realm of ISR, where ML and DL methodologies converge to unravel the intricate patterns inherent in neural signals. This section delves into the diverse strategies and techniques employed for feature extraction, aiming to capture the characteristics of inner speech representations. From traditional signal processing methods to advanced neural network architectures, the methodologies discussed herein underscore the significance of robust feature extraction in enhancing the interpretability and discriminative power of ISR systems.

Gasparini et al. (2022) employed Power Spectral Density (PSD) as a precursor to classification, utilizing Welch’s method for its calculation. The PSD analysis focused on relative power within specific frequency bands, namely alpha (8–13 Hz), beta (13–30 Hz), and gamma (30–100 Hz). Meanwhile, Wang et al. (2013) conducted an experiment with eight participants who mentally read two Chinese characters representing “left” and “one.” Notably, they successfully differentiated between these characters and the resting state. In their study, the feature vectors of EEG signals were extracted through Common Spatial Patterns (CSP).

In contrast, Kim et al. (2014) focused on three specific vowels—/a/, /i/, and /u/. They employed multivariate empirical mode decomposition (EMD) and CSP for feature extraction, coupled with linear discriminant analysis, achieving an accuracy level of approximately 70%. Nieto et al. (2022) employed a CSP filter for each frequency band, from which the average power in the first six spatial components was computed. This process resulted in a 36-dimensional feature vector, comprising six features for each of the six bands, which was utilized for subsequent classification. To ensure uniformity, each feature in the vector was scaled within the range of 0 to 1. Feature extraction of Arjestan et al. (2016) involves the use of CSP filters and EMD. These methods were primarily chosen for their ability to extract discriminative spatial and frequency features from noisy multichannel EEG data. CSP displayed effectiveness in maximizing the variance between inner speech classes, whereas EMD decomposes the EEG signal into intrinsic mode functions that may correspond to temporally meaningful components such as mental syllables or phonemes (Kim et al., 2014; Arjestan et al., 2016). The classification is performed using SVM with a radial basis function (RBF) kernel.

Neural activity and stimulus features contribute to the extraction of input and output features, respectively, in decoding models. Common examples of speech representations for decoding encompass auditory frequencies, modulation rates, or phonemes in the context of natural speech. Neural representations often involve extracting features such as firing rates from single-unit spiking activity or amplitudes in specific frequency bands, such as the high gamma band, from recorded electrophysiological signals.

### 7.2 Classification

The classification stage in the domain of ISR marks a pivotal phase where ML and DL methodologies converge to decipher and categorize the extracted features. As we navigate through the landscape of classification, the emphasis is on unveiling innovative strategies that bridge the gap between the intricate nature of inner speech and the computational frameworks designed to decode its meaningful content. In this review, we assessed a diverse range of ML models that have been applied to ISR. These include traditional approaches, such as support vector machines (SVMs), random forests, and regularized linear discriminant analysis (RLDA), as well as deep learning models, like convolutional neural networks (CNNs), EEGNet, and recurrent architectures like LSTMs and GRUs. These models were selected based on their prevalence in ISR literature and their relevance to the distinct challenges often posed by ISR.

The traditional models offer benefits like simplicity, lower computational costs, and clearer interpretability (Golpour et al., 2020). These advantageous features are deemed important in ISR scenarios when datasets are small, preprocessing is well-optimized, and explainability is a priority. However, they rely on classical statistical learning techniques and make predictions based on the patterns found in manually selected features from the data (Cooney et al., 2019). This drawback and reliance on manual selection can limit their ability to capture the non-linear and distributed patterns often present in neural signals (Li et al., 2019). Consequently, the practicality of traditional models is constrained and limited, hindering their ability to generalize across the high-dimensional and temporally dynamic nature of neural data.

**Figure 3:**
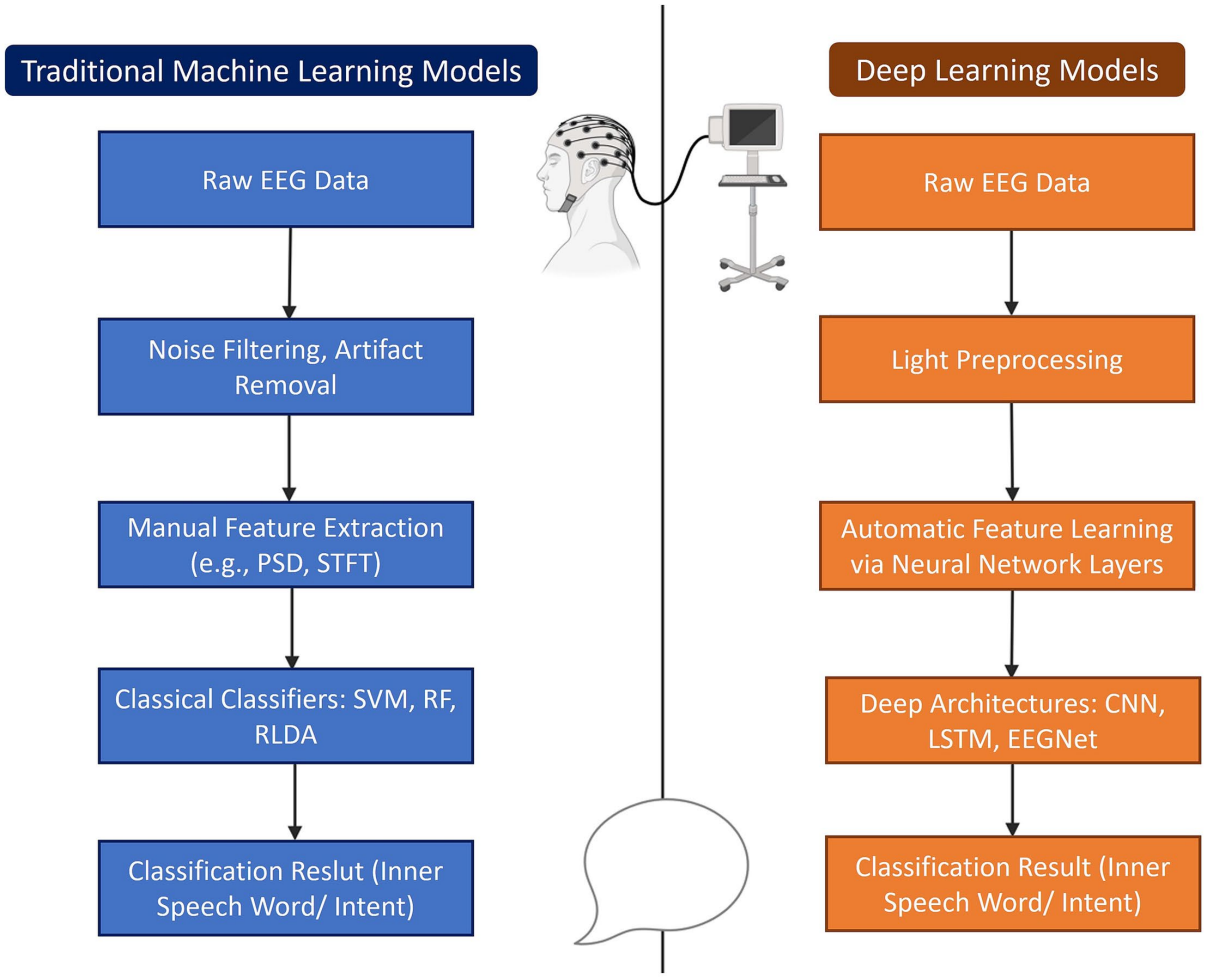
Comparison of traditional machine learning and deep learning approaches for inner speech decoding. Both approaches aim to decode inner speech words or intent from raw EEG data. The traditional pipeline involves extensive preprocessing, manual feature extraction (e.g., Power Spectral Density (PSD), Short-Time Fourier Transform (STFT)), and classification using classical models like Support Vector Machines (SVM), Random Forests (RF), or Regularized Linear Discriminant Analysis (RLDA). In contrast, deep learning models require only light preprocessing, automatically learn features through neural network layers, and utilize deep architectures such as Convolutional Neural Networks (CNN), Long Short-Term Memory (LSTM), or EEGNet for classification.

As opposed to this, DL models, particularly CNNs and recurrent networks, can automatically learn spatiotemporal features from raw data (Lawhern et al., 2018; Lee et al., 2021a). This makes them better suited for modeling the complexity of inner speech, especially when large and high-quality datasets are available. Nevertheless, these models require more data, are computationally intensive, and can often lack transparency (Yousef and Allmer, 2023). Thus, this can be a drawback for clinical applications, especially in ISR given the covert and variable nature of inner speech. Overall, the distinction in the capabilities of the different models has been displayed in multiple studies evaluating their performance. The differences in these processes are illustrated below in Figure 3.

Cooney et al. (2019) have significantly contributed to the decoding of EEG signals for inner speech, particularly evident in their comprehensive evaluation of hyperparameters for EEG classification. Through extensive trials, the authors identified optimal hyperparameters for the Shallow CNN, Deep CNN, and EEGNet. The optimal performance of Shallow and Deep CNNs was achieved using the LeakyReLU activation function, while EEGNet demonstrated superior results with the exponential linear unit (ELU). In addition to assessing these modern approaches, the study included a comparative analysis with established methods, including SVM, Random Forests, and RLDA. Notably, the CNNs outperformed these traditional methods, underscoring the efficacy of convolutional neural networks in the context of EEG signal classification.

Schirrmeister et al. (2017) introduced both deep and shallow CNN architectures designed specifically for EEG signals, while (Lawhern et al., 2018) proposed the EEGNet architecture. These architectures share a common foundation, employing spatial and temporal convolutions to discern patterns and features within the temporal and spatial dimensions, contributing to the effective analysis of EEG data. The classification framework of Lee et al. (2021a) was designed with convolution layers and separable convolution layers to capture temporal, spectral, and spatial information from raw EEG signals. The architecture took raw signals as input (C × T), where C represents the number of channels, while T represents the time dimension. Essentially, the framework aims to classify signals into 9 speaker classes.

CNN architectures are often selected due to their alignment with previously successful methods (Singh and Gumaste, 2020). These established methods typically involve the initial identification of feature vectors, which are then used to train a classifier. CNNs follow a similar procedure by first extracting features through convolutions and subsequently utilizing them for classification. An inherent advantage of CNNs is their ability to concurrently train both feature extraction and classification.

On the other hand, for inner-overt speech decoding in the study conducted by Martin et al. (2018), a linear mapping model was employed, representing the speech features (spectrogram or modulation) as a linear weighted sum of neural activity at each electrode. The model parameters were determined using gradient descent with early stopping regularization. The data were divided into training and testing sets, and model fitting was performed with a jackknife resampling technique. The algorithm monitored out-of-sample prediction accuracy and terminates after a specified number of iterations.

**Figure 4:**
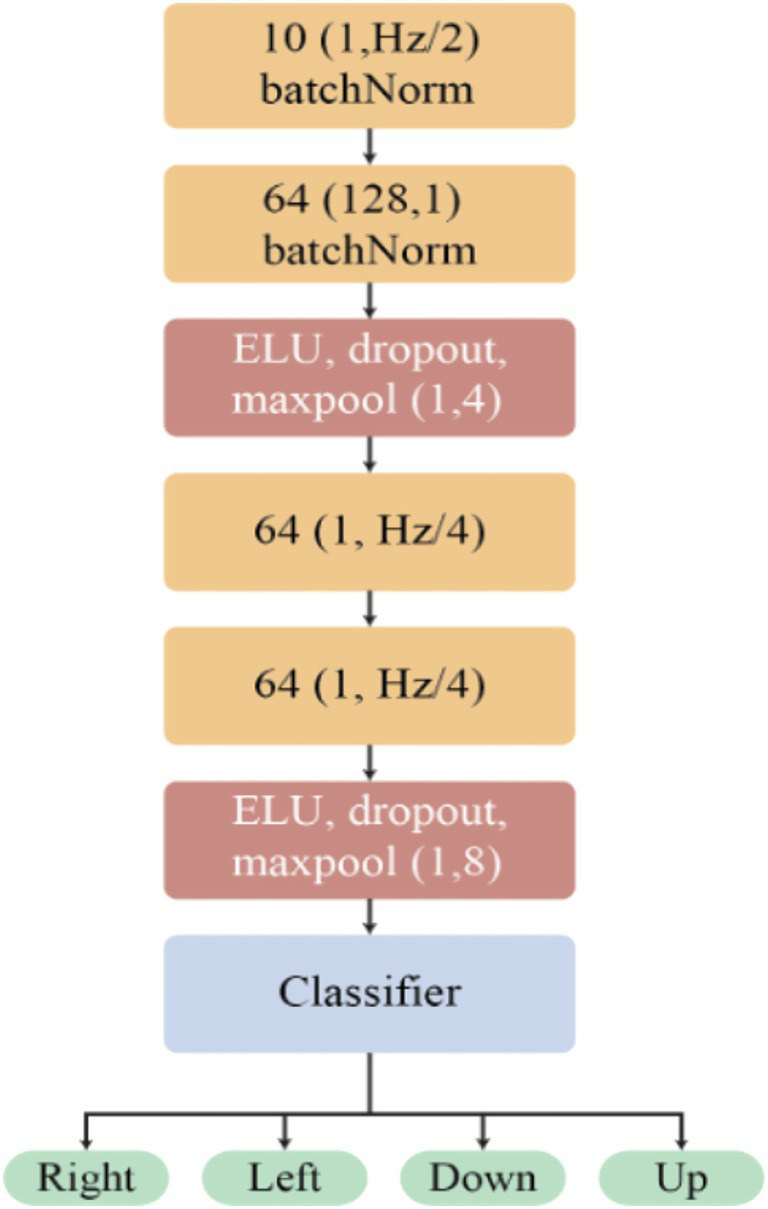
Architecture of the EEG-based classification model. The model begins with two batch normalization layers, processing 10 and 64 input features, respectively. Subsequent layers involve Exponential Linear Unit (ELU) activation, dropout for regularization, and max-pooling operations (1,4) and (1,8) to down sample the features. The core of the network consists of convolutional layers with 64 filters, designed to extract relevant patterns from the EEG signals. Finally, a classifier layer processes the learned features to predict one of four distinct output classes: right, left, down, or up.

The EEGNet model stands out as a widely adopted architecture for inner speech classification. Its design incorporates principles akin to those found in Shallow and Deep CNNs, particularly concerning temporal and spatial convolutions (Jonsson, 2022). Consequently, the initial two convolutional layers exhibit similarities to the corresponding layers in these architectures, albeit with slight variations in the number of filters and the kernel size of the temporal convolution, which is contingent on the data’s sample rate. Notably, EEGNet introduces an enhancement in the form of a depth-wise separable convolution (Chollet, 2017), discernible in convolution layers three and four as illustrated in Figure 4.

The exploration of classification methodologies in the context of ISR provides valuable insights into the diverse approaches and architectures employed to discern patterns within EEG signals. From the widely adopted EEGNet model to the configurations of Shallow and Deep CNNs, the classification segment has highlighted the evolution of techniques for effectively categorizing inner speech representations. The utilization of convolutional layers for temporal and spatial feature extraction has proven to be instrumental, offering a robust foundation for classification models. Furthermore, the integration of depth wise separable convolutions, as exemplified by EEGNet, demonstrates the ongoing refinement and innovation in enhancing the discriminative power of these models (Lawhern et al., 2018). As the field progresses, the continual exploration and integration of advanced classification techniques promise to propel the capabilities of ISR systems, fostering a deeper understanding of the complex interplay between neural signals and the inherent intricacies of spoken language.

## 8 Performance evaluation of ISR models

This section provides a comparative overview of the performance metrics reported in important ISR studies. We assess the relative advantages and disadvantages of the existing ISR methodologies by synthesizing the classification accuracies, sample characteristics, and model types.

Recent studies show a broad spectrum of classification accuracies, which are largely affected by the model type, dataset size, and complexity of the speech class. CNN-based models, such as EEGNet, have demonstrated greater accuracy in classifying limited word sets than traditional methods like SVM or Random Forest. According to research conducted by Simistira Liwicki et al. (2022), a customized iSpeech-CNN reached an accuracy of 29.04% over 11 classes and had an F-score of 36.18%. In contrast, Berg et al. (2021) noted that the accuracy of 2D-CNN for 4-class inner speech word classification was 29.67%, which is just a bit higher than chance level.

Moreover, research such as that by Kiroy et al. (2022) has shown that multilayer perceptrons (MLPs) yield better performance than SVMs when it comes to classifying spatial directional words in inner speech, with accuracy reaching as high as 47.3% in 6-class scenarios. Conversely, models that are trained on small subject pools or complex class sets (e.g., Shepelev et al., 2021 with emotional intonations) tend to exhibit greater variability.

In particular, investigations employing ECoG or high-resolution fMRI (e.g., Martin et al., 2018); Although Jones and Voets (2021) achieve significantly higher decoding accuracy, their work does not lend itself to real-time application. The results underscore a trade-off between decoding fidelity and practical deployment.

These findings are summarized in Table 3, which compares the model type, dataset, number of classes, evaluation metrics, and performance scores. The necessity for scalable models, solid data preprocessing, and benchmark datasets for equitable comparison is underscored by these results.

**Table 3:** Inner speech classification procedures, evaluation metrics and results.

Article	Procedure	Evaluation metrics	Results	Sample size	Class set size	Public availability
Simistira Liwicki et al. (2022)	The authors employ a tuned i-Speech CNN architecture for the classification of five vowels and six words using a publicly available dataset.	Precision	29.04	15	11 (5 vowels: /a/, /e/, /i/, /o/, /u/; 6 words: arriba/up, abajo/down, derecha/right, izquierda/left, adelante/forward, atrás/backwards)	Yes (available at: https://github.com/LTU-Machine-Learning/Rethinking-Methods-Inner-Speech)
		Weighted F-score	36.18			
		F-score	21.84			
Berg et al. (2021)	Employing a 2D Convolutional Neural Network (CNN) modeled on the EEGNet architecture, the researchers categorized EEG signals from eight subjects during internal contemplation of four distinct words.	Accuracy	29.67	8	4 words (up, down, left, right)	Yes (available at: https://doi.org/10.18112/openneuro.ds003626.v2.1.0)
		Precision	29.76			
		Recall	29.68			
		F1-score	29.61			
Jones and Voets (2021)	The authors trained phoneme-level decoders on a large, elicited inner speech dataset in a single subject. A second self-generated inner speech dataset was obtained from the same subject. Despite being trained solely on elicited inner speech neural recordings, the decoders accurately predicted unseen phonemes in both test conditions.	Median test accuracy (Transfer analysis)	47	1	9 target syllables (/ga/, /gi/, /gu/, /ma/, /mi/, /mu/, /sa/, /si/, /su/)	Not available
		Median test accuracy (Replication analysis)	50.82			
Kiroy et al. (2022)	Investigated were values recorded from 14 channels of 10 young men engaged in real verbalization (spoken speech) and the pronunciation of imagined words signifying directions in space (up, down, right, left, forward, backward).	SVM accuracy	43.7	10	6 words (up, down, right, left, forward, backward)	Not available
		MLP accuracy	47.3			
Nalborczyk et al. (2020)	The authors report findings from a preregistered experiment examining the electromyographic correlates of overt and inner speech production for two phonetic classes of nonwords. An automatic classification approach discerned articulatory features in nonwords during both overt and covert speech.	Mean	59.70	25	20 nonwords were used: 10 rounded and 10 spread nonwords	Yes (available at: https://osf.io/czer4/)
		SD	60.09			
		Median	42.03			
Shepelev et al. (2021)	Two training approaches for the models were proposed and evaluated. The impact of parameters on mel-frequency cepstral coefficients calculation was investigated to understand its influence on resultant accuracies.	Accuracy	80	12	3 classes: Confident (C), Uncertain (U), Neutral (N)	Not available
Stark et al. (2017)	Scores for inner speech (categorized by group) were correlated with specific language and cognition measures extracted from the comprehensive aphasia test.	Mean	64	38	3 classes: Relatively preserved, preserved, and unclassified inner and overt speech	Yes (available at: https://doi.org/10.23641/asha.5303542)
		SD	13			
Arjestan et al. (2016)	Common spatial patterns (CSP)	Energy, variance, ZCR, skewness, and kurtosis	81.3	5	3 syllables (/kaː/, /fiː/, /suː/), 6 vowels (/æ/, /e/, /au/, /aː/, /iː/, /uː/), resting (Persian)	Not available
Lee et al. (2021b)	Temporal changes	Root mean square	76.19	9	9 subjects (speaker ID)	Not available

It is crucial to remember that results from different research cannot be directly compared with regard to the performance measures in Table 3 because of differences in sample sizes, recording modalities, class sets, and preprocessing methods. While most original research did not include formal statistical comparisons (e.g., *p*-values, confidence intervals), metrics such as standard deviation or median values (e.g., Jones and Voets, 2021; Nalborczyk et al., 2020) provide some sense of variability when available. Therefore, when analyzing performance patterns across different datasets, care should be used.

## 9 Technical limitations and future directions

Addressing ISR’s fundamental drawbacks is essential to enhancing performance and permitting wider use as it develops, particularly in BCI frameworks. In addition to outlining possible research directions to lead future innovation, this part offers a cohesive examination of technological restrictions.

- ***Signal Quality and Noise***
  A core difficulty in ISR is the low SNR of neural recordings, especially in the case of EEG. Subtle in nature, inner speech signals can be affected by muscle artifacts, eye blinks, and ambient noise. Methods like independent component analysis (ICA), adaptive filtering, and frequency band optimization have been extensively employed to enhance signal clarity (Mullen et al., 2015; Craddock et al., 2016; Cohen, 2017; Zhang et al., 2022). Yet, it is still challenging to fully isolate inner speech signals. Hybrid modalities and multimodal sensing methods present possible avenues for progress (Liu and Ayaz, 2018; Wellington et al., 2024). According to Pei et al. (2011), intracranial methods like ECoG have specifically shown improved signal fidelity and increased decoding accuracy. Despite their intrusive nature, these methods demonstrate the limits of ISR decoding capabilities in high-SNR settings.
- ***Generalization and Inter-Subject V*a*riability***

Individual differences in brain representations of inner speech make it difficult for ISR models to generalize across users. This heterogeneity is caused by a variety of factors, including inner speech formulation styles, brain structure, and language habits (Stark et al., 2017; Martin et al., 2018; Perrone-Bertolotti et al., 2014). Cross-subject model transferability is difficult as a result. This can be lessened via domain adaptation and transfer learning, which reuse existing information to adjust to new users (Wilroth et al., 2023). Usability in actual BCI environments may be further enhanced by customized calibration procedures.
***Benchmarking and Dataset Limitations***

Reproducibility and model robustness are hampered by the dearth of sizable, varied, and publicly accessible ISR datasets. The lack of subjects, speech classes, or modalities in many of the current datasets makes comparison and generalization challenging (Nieto et al., 2022; Shepelev et al., 2021). Benchmark datasets with diverse people, languages, and situations are desperately needed (Cannard et al., 2024; Kaur et al., 2020). Additionally, uniform evaluation procedures and open-source pipelines will improve cross-study comparability (Lotte et al., 2013).
***Clinical Trust and Interpretability***

The clinical and user acceptability of deep learning models is limited since they frequently function as “black boxes.” Building confidence and guaranteeing model reliability depend on interpretability. The integration of explainable AI (XAI) techniques like as SHAP and LIME has been emphasized in recent work in ISR and more generally in medical AI (Ribeiro et al., 2016; Lin et al., 2023; Gandin et al., 2021). In high-stakes situations like assistive communication, these techniques can facilitate transparent decision-making and aid in the visualization of feature contributions.
***Neurosecurity, Privacy, and Ethics***

Serious ethical questions about privacy, autonomy, and possible abuse of cognitive data are brought up by the decoding of inner speech. Since neural data is extremely private, there are significant hazards associated with its improper interpretation or preservation (Yuste et al., 2017). To avoid abuse and advance user safety, ethical ISR development must incorporate informed permission, data anonymization, and institutional control (Masters, 2023; Lee et al., 2021a).
***Real-time applications with multimodal integration***

Future ISR systems should use multimodal techniques, integrating brain inputs with physiological or behavioral indicators like eye tracking, facial movements, or GSR, to improve performance and contextual awareness (Selfridge et al., 2011). This kind of integration might make it easier to distinguish irrelevant mental activity from inner speech. Real-time ISR applications in clinical and consumer-facing settings can be made easier by developments in portable EEG, dry electrodes, and low-latency processing architectures. (Lawhern et al., 2018; Minguillon et al., 2017)

## 10 Conclusion

This review has highlighted the pivotal role of machine learning (particularly models like CNNs and EEGNet) in advancing the domain of inner speech recognition (ISR). By analyzing the various key components across the ISR pipeline, from neural signal acquisition to preprocessing and model architecture, we outlined how ML allows for more accurate, robust and scalable decoding of inner speech. Moreover, our proposed structured framework offers a practical guide for improving ISR performance, setting the stage for future innovation.

In conclusion, despite the challenges facing ISR, including issues of interpretability, ethical concerns, and dataset diversity, the future of ISR remains promising. Technological advancements in machine learning and neuroimaging, along with deeper insights into the relationship between inner speech and brain dynamics, will drive the field forward. By addressing these limitations through collaboration, standardized criteria, and improved neuroimaging techniques, ISR systems can become more effective, inclusive, and responsible. The synergy between machine learning and cognitive neuroscience not only enhances technology but also advances our understanding of the profound mechanisms underlying inner speech, paving the way for a transformative era in ISR research.
